# Anal Fissure and Its Treatments: A Historical Review

**DOI:** 10.3390/jcm13133930

**Published:** 2024-07-04

**Authors:** Cristiana Riboni, Lucio Selvaggi, Francesco Cantarella, Mauro Podda, Salvatore Bracchitta, Vinicio Mosca, Angelo Cosenza, Vincenzo Cosenza, Francesco Selvaggi, Bruno Nardo, Francesco Pata

**Affiliations:** 1 Department of Pediatric Surgery, UKBB (Universitäts-Kinderspital beider Basel), Spitalstrasse 33, 4031 Basel, Switzerland; riboni.cristiana01@gmail.com; 2 Department of Advanced Medical and Surgical Sciences, Università degli Studi della Campania “Luigi Vanvitelli”, 80138 Naples, Italy; vinicio.mosca@unicampania.it (V.M.); angelo.cosenza@unicampania.it (A.C.); francesco.selvaggi@unicampania.it (F.S.); 3 CPEP (Centre for Proctology and Perineology), Ospedali Privati Forlì, 47121 Forlì, Italy; fcanta81@gmail.com; 4 Department of Surgical Science, Università degli Studi di Cagliari, 09042 Cagliari, Italy; mauro.podda@unica.it; 5 Coloproctology Centre, Clinica del Mediterraneo, 97100 Ragusa, Italy; dott.salvatorebracchitta@gmail.com; 6 Plastic and Reconstructive Surgery Unit, Multidisciplinary Department of Medical-Surgical and Dental Specialties, Università degli Studi della Campania “Luigi Vanvitelli”, 80138 Naples, Italy; vincenzocosenza01@gmail.com; 7 Department of Pharmacy, Health and Nutritional Sciences, University of Calabria, 87036 Rende, Italy; bruno.nardo.editor@gmail.com (B.N.); francesco.pata@gmail.com (F.P.); 8 A.O. Annunziata, 87100 Cosenza, Italy

**Keywords:** anal fissure, history, historical overview, proctology, history of surgery, fissure-in-ano

## Abstract

Anal fissure is one of the most common proctological pathologies. It consists of the formation of a longitudinal tear in the anoderm, causing pain and bleeding during and after defecation. When chronic, it can significantly negatively impact the quality of life of the affected patient. Currently, multiple therapeutic options are available, both medical and surgical. The objective of this article is to highlight the historical evolution in the physiopathological understanding and treatment of this disease, underlining the key moments in this history. This is the first article to summarize the milestones in the treatment of anal fissure from ancient to current times.

## 1. Introduction

With a lifetime risk of experiencing an episode of 7.8%, as estimated in the US population, anal fissure (fissure-in-ano) is one of the most common proctological disorders [1]. This is characterized by intense pain during and after defecation, bleeding, itching, and sphincter hypertonicity. The fissure consists of a linear split in the anoderm that can extend from the dentate line to the anocutaneous margin. It is located in the posterior midline in 80–90% of cases, and in the remaining cases, it can be found either anteriorly or both anteriorly and posteriorly. It is typically triggered by the passage of hard stools, which determines the initial longitudinal tearing; subsequently, due to the induced pain, it establishes hypertonicity of the sphincter, which favors the reopening of the fissure during subsequent defecations [2]. This phenomenon is defined as “acute anal fissure” and tends to resolve spontaneously, or after therapy, within 6 weeks. When this does not occur, the anal fissure becomes chronic and healing becomes more challenging [3]. The chronic form may also involve the presence of a sentinel pile or hypertrophied anal papilla. Several medical and surgical treatments have been proposed throughout history.

The aim of this article is to describe the evolution of medical and surgical anal fissure therapy through the centuries, highlighting the crucial steps and major contributors in this field. Based on the level of understanding of the pathology and the progress in treatment, it is possible to distinguish a pre-modern (until the 19th century) and a modern era (until today).

## 2. Pre-Modern Era

**Figure 1:**
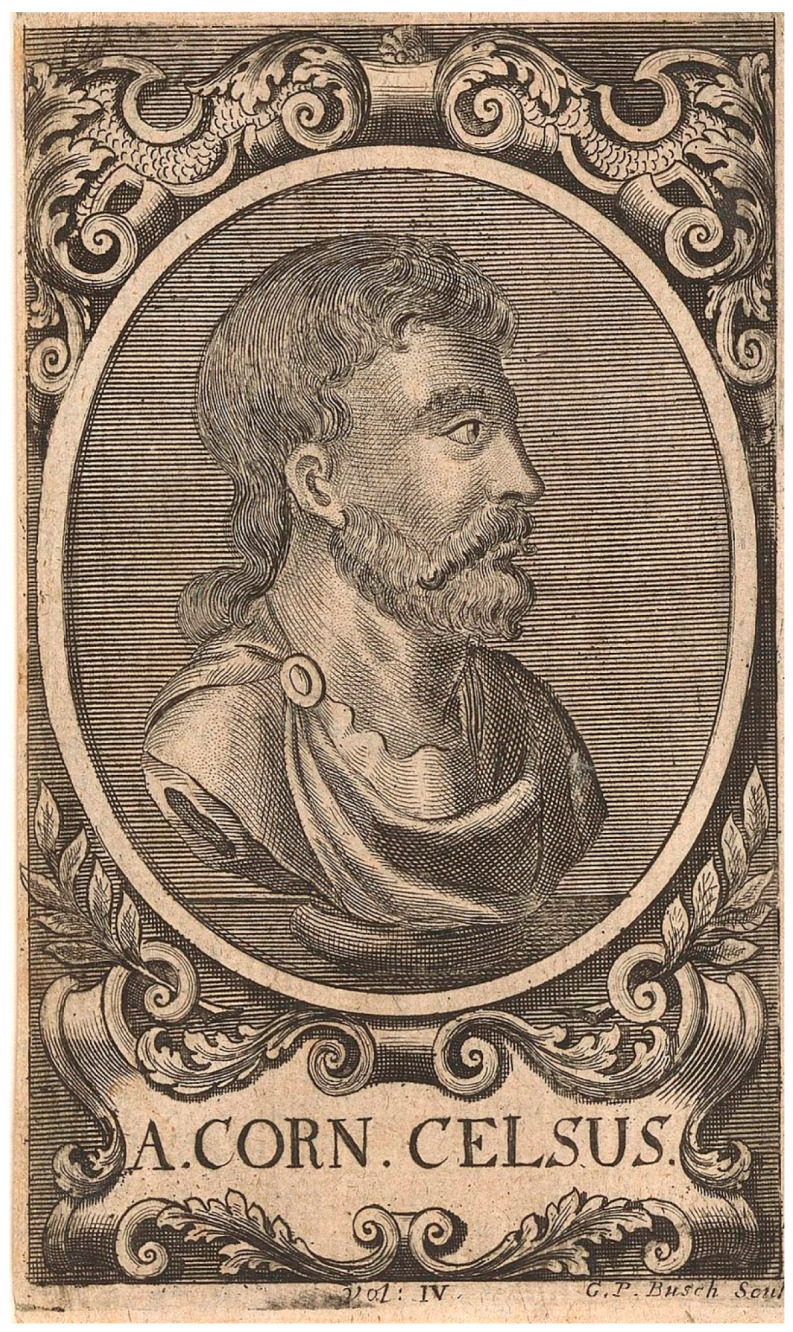
Aulus Cornelius Celsus (Georg Paul Busch, CC0, via Wikimedia Commons, https://upload.wikimedia.org/wikipedia/commons/8/8c/Portret_van_Aulus_Cornelius_Celsus%2C_RP-P-1908-2809.jpg (accessed on 25 June 2024).

The testimonies of anal fissure in ancient history are extremely scarce. Although anal fissure is mentioned in the most important text of ancient Indian medicine, the *Sushruta Samhita*, dating back to the 6th century BC, under the term *Parikartika* [4], the first author to describe anal fissure in detail was the Roman nobleman **Aulus Cornelius Celsus** (25 BC–50 AD) (Figure 1) [5]. In his work *De re medica*, he wrote that the anus could easily damage its skin and he proposed sitz baths as treatment, with hot water alternating with the local application of ointments [6].

Further ancient testimonies on the treatment of anal fissure have come to us from the Persian authors Abū Bakr al-**Razi** (854–925 AD) and **Ibn Sina** (989–1037 AD), who proposed lifestyle changes as the most effective treatment. As a first-line treatment, they suggested a laxative diet (soft-boiled egg yolk, cabbage soup, coconut, almonds, brown sugar) in combination with topical ointments made of animal fats (like those of hen, goose, goat, and camel) and a warm water sitz bath. They also suggested sitting on soft surfaces and avoiding hard ones (e.g., as in horseback riding) and avoiding scratching [7]. As a second-line treatment, they proposed multidrug treatments with herbal extracts and manual manipulation [8].

Probably the first detailed anatomical description of anal fissure belongs to **Louis Le Monnier** from his *Traité de la fistule de l’anus ou du fondement* (*Treatise on the Fistula of the Anus or the Rectum*), published in 1689. During the same period, anal fissure was also described by Arab authors who proposed constipation as the triggering cause; they suggested scraping the fissure with a scalpel or a fingernail in order to create a fresh wound and promote healing [9].

However, although it was described as a painful and debilitating condition, no treatments other than enemas and ointments were widely in use until the 19th century [10].

## 3. Modern Era

### 3.1. 19th Century

Two main surgical practices were developed during the 19th century: dilatation and sphincterotomy in its variants.

Since the early 1800s, the etiology of anal fissure has been debated. Some authors, like **Alexis Boyer** (1757–1833), believed that the cause of fissure was a spasm of the anal sphincter, while others, like **William Holme Von Buren** (1819–1883) and **Joseph McDowell Mathews** (1847–1928), believed that spasm was a consequence of fissure [11].

In 1818, Boyer, the first surgeon of Napoleon, relying on his theory, was the first to propose open lateral sphincterotomy as a treatment for anal fissures, pointing out that the division of the sphincter was followed by the total regression of symptoms [12]. The technique was described as follows: “*Upon a well-oiled finger, a slender bistoury with a narrow blade and a rounded point is inserted into the rectum. The cutting edge be directed either to the right or to the left depending on the location of the anal fissure. The surgeon now separates the intestinal lining, the subcutaneous tissue, and the anal sphincter muscle with one single cut*” [13]. He emphasized that, for a successful operation, the incision should have involved the entire thickness of the muscle (both internal and external sphincter) at is lower portion [11,14]. Boyer published a description of this procedure applied on a 26-year-old woman and reported complete healing without the occurrence of fecal incontinence [12]. **Baron Guillaume Dupuytrens** (1777–1835) later modified the procedure by suggesting cutting only the superficial fibers of the sphincter [14]. However, although we consider the sphincterotomy a routine procedure, at the time, it caused severe complications including pelvic abscesses and death, as reported by **Alfred Velpeau** (1795–1876) in four patients [12].

In 1829, **Joseph Recamier** (1774–1852) was the first to propose anal dilatation as a therapeutic alternative to surgery. This was performed by inserting two fingers into the rectum and then pressing with the thumb on the entire circumference of the sphincter to overcome its resistance [11,15]. The procedure attracted the interest and support of many surgeons. However, it was also criticized because it could not be standardized, and it was unclear how much force had to be exerted to avoid the risk of sphincter injury [15].

Another drawback of the procedure was the need to repeat the procedure several times on the same patient to achieve a result, so **Jules Maissoneuve** (1809–1897) proposed a faster and more complete dilation. This was achieved by introducing the fingers one by one into the rectum until the whole hand was inside it. At that point, the surgeon closed the hand into a fist and withdrew it forcefully. Obviously, in the absence of anesthesia, the procedure was extremely painful. The technique quickly fell into disuse and Maissoneuve himself modified it, using simple stretching with the two index fingers under chloroform administration [11].

In 1835, Sir **Benjamin Collins Brodie** (1783–1862) described the lateral sphincterotomy, finding that this had better results in terms of healing time compared to the posterior sphincterotomy [15].

**Figure 2:**
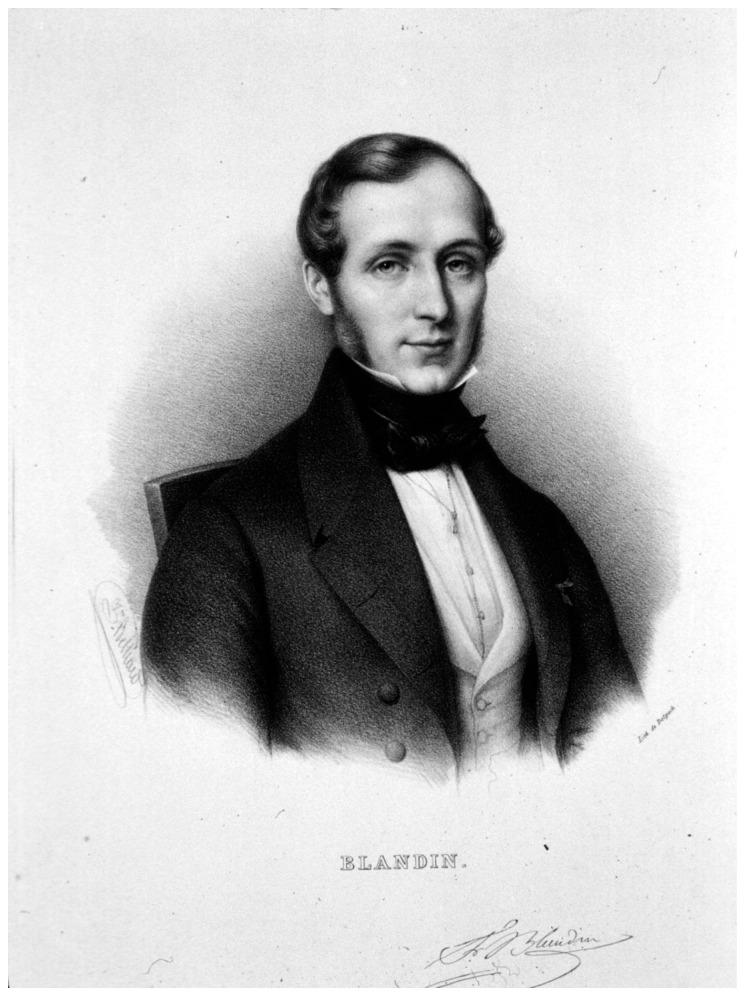
Philippe-Frédéric Blandin (Zéphirin Belliard, public domain, via Wikimedia Commons: https://upload.wikimedia.org/wikipedia/commons/3/31/PF_Blandin.jpg (accessed on 25 June 2024).

**Philippe-Frédéric Blandin** (1798–1849) (Figure 2) first described the procedure of “closed subcutaneous lateral sphincterotomy”. The operation was structured into four surgical stages: (1) prick the skin, (2) insert a finger into the anus and stretch the perianal skin (3), apply a tenotome deep into the anal mucosa, and (4) use the tenotome to dissect the muscle [16]. In order to avoid a lesion of the rectal mucous layer and subsequent fistula formation, **Joseph F. Charrière** (1803–1876) created a modified tenotome with a retractable blade.

**L.A. Delley**, in 1855, supposed that the initial trigger of the longitudinal tear in the perianal skin was the passage of hard feces through the anus. He described the pathology in this way: “*In such cases, emptying the bowels is always associated with stabbing pain which in case of an already persistent anal fissure outlasts the act of defecation itself for several hours or even for days and often radiates into the sacral region. In rare cases, but still frequently enough, there are even incidents of loss of consciousness and antiperistaltic movements of the stomach. As a consequence, people afflicted with anal fissure try to delay this painful act as long as possible by deliberately contracting the anal sphincter muscle to reverse defecation for some hours*” (translated from German) [17]. This phenomenon also favors constipation and, consequently, was identified as the basis of the pain–constipation–pain cycle. Delley divided fissures into three stages: linear erosion, fresh longitudinal tear (in the eroded mucous membrane), and ulcerous form. This staging was the precursor of the modern classification of acute and chronic fissure [10].

In 1835, **Frederick Salmon** (1796–1868) founded the “Benevolent Dispensary for the Relief of the Poor Afflicted with Fistula, Piles and other Diseases of the Rectum and Lower Intestines”, which was the first institution committed to the treatment of anorectal disease. In 1853, it moved to Charterhouse Square to allow more beds and was renamed “St Mark’s Hospital for Fistula and other Diseases of the Rectum” [18,19,20].

This institution was visited by many eminent surgeons, including **Joseph McDowell Mathews** (1847–1928), who practiced there with **William Allingham** (1829–1908) in 1878 and, subsequently, founded the American Proctologic Society in 1899 (renamed the American Society of Colon and Rectal Surgeons in 1973), being considered the “*Father of American Proctology*” [21].

At the end of the 19th century, McDowell analyzed the methods developed by his colleagues and, based on his experience, concluded that dilation should be the first-choice method. Only if dilation could not be performed should sphincterotomy (of both the external and internal sphincter) be attempted. He emphasized the importance of performing a sphincterotomy only superficially, as the involvement of deep fibers was often associated with incontinence. He believed that only a small part of the muscle fibers was involved in the ulcer, and that by resting them by dilatating the entire sphincter, the ulcer could heal. To confirm his theory, he reported the case of a gentleman who had been examined by him because of anal pain after defecation. Dr McDowell had performed the examination using a speculum and had identified an anal fissure. However, the patient refused all treatments as he had to leave the city. Shortly afterwards, the doctor received a letter in which the gentleman stated that he had been cured by the examination itself. Dr McDowell underlined in his book that he did not send the patient a bill until he received this letter. A similar event happened to Dr Curling [11].

### 3.2. 20th Century

In this era, medical therapy (based on laxatives, topical emollients, and warm sitz baths) continued to be the most widespread approach for the acute fissure, especially due to its spontaneous tendency to heal [15].

In the 20th century, the practice of sphincterotomy slowly became established as the first surgical choice in the treatment of chronic anal fissure, surpassing dilation, as reported by **Ottomar Rosenbach** (1851–1907), who (in 1900) described “*forced dilatation or dissection of the anal sphincter muscle as cruel, hardly calculable surgery, which at best can be granted the right of existence only as the last and heroic resource*” [10].

In fact, **Samuel T. Earle (1849–1931)**, President of the American Proctologic Society in 1902–1903, claimed that the most common practice for the treatment of anal fissure in the early 1900s was dilatation, at least among those who were not specialists in proctology. This was because proctologists, being in contact with their patients and having to treat those who had been treated unsuccessfully by other specialists, noticed that this method had the highest number of recurrences. This is why he recommended excision of the fissure, which was then left to heal by granulation [22].

In 1914, **Louis Jacob Hirschmann** (1878–1965) first proposed the anal dilatation method using instrumental dilators with a fixed diameter [10,23].

In 1930, **William Bashall Gabriel** (1893–1975) suggested the excision of the fissure in association with posterior sphincterotomy. Although this technique had a high healing rate, it frequently caused a “keyhole deformity” and was totally abandoned [24,25].

**W.B. Gabriel**, in 1929, and **Clifford Naunton Morgan** (1901–1986), in 1935, both from St. Mark’s Hospital, hypothesized that the primary cause of the chronicity of anal fissures was sphincter spasm resulting from pain. Observing the promising outcomes of pruritus ani treatment with local anesthesia, they proposed that anal fissures could be conservatively treated through injections at the level of the external anal sphincter and the base of the fissure using a combination of A.B.A. (anaesthesin, benzyl alcohol, and olive oil) and benacol (para-amino-benzoyl-benzoate, phenmethylol, and almond oil) [26,27]. However, **Harry Bacon** (1900–1981) reported that these injections caused local discomfort in his patients and often increased sphincter tone [26,28]. Additionally, it was later highlighted that healing occurred in less than half of the patients, with frequent complications such as infection, discomfort, and incontinence. Consequently, this treatment was rapidly abandoned [15].

In 1939, **William Ernest Miles** (1869–1947) illustrated the “pectenotomy” as the procedure of choice for the treatment of idiopathic anal fissures. This consisted of the division of the “pecten band”, a structure identified in 1918 by Miles himself, who described it as a purely pathological fibrous deposit that formed in the lower part of the anal canal as a result of congestion due to sphincter spasm. However, it was later pointed out by Eisenhammer that this line would be nothing more than the lower margin of the hypertonic internal sphincter [29,30].

**Figure 3:**
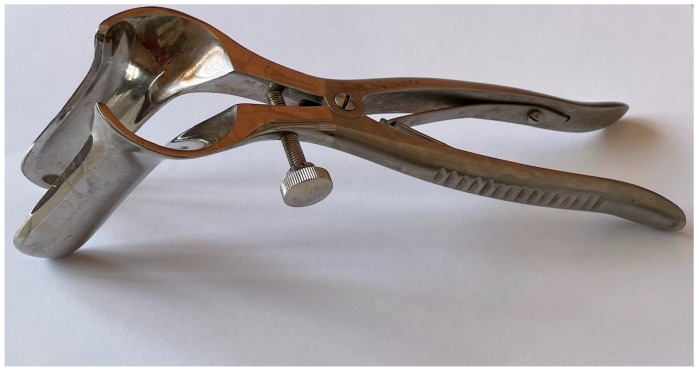
Eisenhammer’s self-retaining rectal speculum (courtesy of the Department of Advanced Medical and Surgical Sciences, University of Campania “Luigi Vanvitelli”, Naples, Italy).

**Stephen Eisenhammer** (1906–1995) himself clarified the anatomy behind sphincterotomy, a practice that had been common since the 19th century, but which was believed to involve the external anal sphincter [15,29,31]. In 1951, in fact, he explained the anatomy of the anal canal in detail and stated that it was the internal anal sphincter that controlled the greatest possible degree of dilatation. Its chronic contracture would also have been responsible for the onset of anal fissures. The effectiveness of the intervention would, therefore, be linked to the resection of the fibers of the internal anal sphincter, and based on this, Eisenhammer described the internal anal sphincterotomy combined with the resection of the hardened margin of the fissure and the sentinel nodule in order to achieve faster healing [15,31,32,33]. He also specified later that lateral sphincterotomy had fewer functional complications than posterior sphincterotomy, while Morgan and Thompson standardized posterior internal anal sphincterotomy in 1956. Eisenhammer further perfected the anal speculum necessary for this procedure, developing the anal speculum most commonly used nowadays in proctologic surgery (Figure 3).

In 1962, **Richard C. Bennett** (1930–2018) and **John C. Goligher** (1912–1998), while confirming the effectiveness of Eisenhammer’s operation, pointed out two drawbacks: the wound took several weeks to heal, and the operation was associated with a number of minor anal continence defects. Therefore, they decided to treat the anal fissure by simply stretching the sphincter so to avoid creating wounds. However, this practice was associated with a high incidence of persistence or recurrence of the fissure itself [29]. Bennett and Goligher, consequentially, after having published their results obtained with stretching and with sphincterotomy in two papers, reached the conclusion that the first procedure was ideal for new diagnoses and the second for relapses [34,35].

In the 1960s, in order to avoid a large wound and the already-pointed-out complications of the postero-medial internal anal sphincterotomy procedure, some authors reintroduced and enhanced subcutaneous sphincterotomy, an idea already presented in 1846 by **Jean N. Demarquay** (1814–1875) and later in 1923 by **Edward Martin** (1859–1938) [15,31,36].

**Figure 4:**
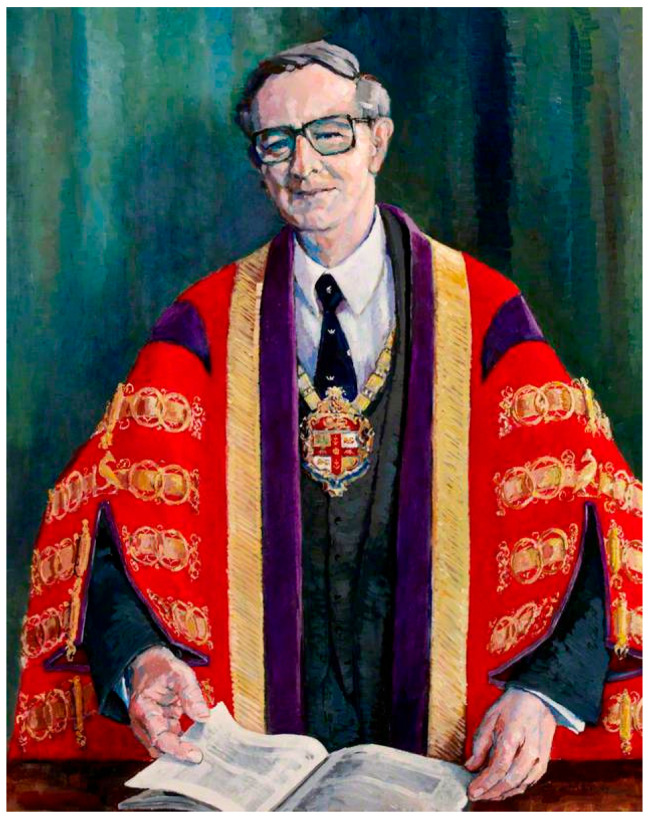
Sir Alan G. Parks (1920–1982) (John Peter Blandy 1927–2011, Hunterian Museum, The Royal College of Surgeons of England).

In fact, to address the same problems highlighted by Bennett and Goligher, in 1967, Sir **Alan G. Parks** (1920–1982) (Figure 4) suggested performing a lateral sphincterotomy through a short circumferential incision in the skin outside the lateral anal margin, suturing it after the operation was completed [15,29,37]. The advantage of this technique, as highlighted by Hawley (1969), was to avoid the presence of a wound in the anal canal [29].

In 1968, **Mitchel J. Notars** (1933–2011) described the lateral subcutaneous internal sphincterotomy, which consisted of sectioning the lower part of the internal sphincter subcutaneously, leaving virtually no external wounds. This method became rapidly popular and was used as the first-choice method in the following years [29,31,32,36].

In 1969, **Peter Lords** (1925–2017) introduced the so-called “Lords procedure” for the treatment of hemorrhoids, which consisted of anal dilation using four fingers of each hand while the patient was sedated. This procedure was also used to treat anal fissures, but was gradually abandoned due to the risk of injury to the internal sphincter and subsequent incontinence [19,32].

In 1970, **Ralph B. Samson** and **William R. C. Stewart** introduced a skin flap as an alternative to lateral sphincterotomy to cover the skin defect of the fissure and promote healing [38]. In the following years, this method was used as an adequate alternative, especially for patients who had already undergone anal surgery or trauma, or patients who had normal or reduced sphincter pressure and, in general, for patients who would be at high risk of incontinence after sphincter surgery [15,39,40].

As already mentioned, by the mid-1970s, the procedure recognized as the gold standard was sphincterotomy. The progressive establishment of the latter was highlighted in Goligher’s book *Surgery of the Anus, Rectum and Colon* (1967) in which he stated that the first line for the treatment of anal fissures was sphincter stretching, but in 1975, he asserted that “*Lateral subcutaneous internal sphincterotomy is now my preferred operation for the treatment of idiopathic anal fissure*” [41,42].

However, in 1992, **Norman Sohn** felt that the superiority of sphincterotomy over dilatation still needed to be questioned because sphincterotomy would be associated with several complications related to the operative wound. Furthermore, trials comparing the two procedures showed conflicting results, suggesting that the effect on fissure healing would be comparable; however, dilatation would be associated with fewer complications. The author understood that the criticism against dilatation was mainly based on the non-reproducibility of the procedure, and he pointed out that this was variable from surgeon to surgeon, but also from patient to patient. To overcome this limitation, he developed a precise and reproducible procedure with the Parks’ retractor opened to 4.8 cm or with a 40 mm rectosigmoid balloon and considered that it should be the new gold standard for the treatment of anal fissures [41].

A different approach to sphincterotomy, with the aim of minimizing postoperative complications, was proposed by **David R. G. Littlejohn**, who reviewed a series of 352 patients undergoing tailored lateral sphincterotomy between 1976 and 1996. This procedure consists of sectioning the internal anal sphincter up to the superior limit of the fissure, and not at the dentate line [43]. This procedure, as confirmed by subsequent studies [44,45,46], has been shown to be similar to traditional LIS in terms of effectiveness; however, an advantage of tailored LIS in terms of continence has not been unequivocally confirmed [47].

In 1993, **Wolfgang H. Jost** and **Klaus Schimrigk** first described the use of botulinum toxin for the treatment of anal fissure associated with increased sphincter tone [15,48,49]. The rationale was based on the fact that the toxin inhibits the release of acetylcholine and causes muscle paralysis. The toxin itself had already been used for the treatment of various diseases associated with muscle hypertonicity (e.g., spasmodic torticollis or achalasia) [15]. On the basis of this information, the toxin was considered a viable alternative to surgery. It reduces internal anal sphincter tone for about 2–3 months, allowing the fissure to heal, with the advantage of avoiding a permanent injury of the internal sphincter [48,49].

Current guidelines suggest the use of botulin toxin injection as alternative first-line therapy, or second-line therapy after unsuccessful treatment with topical nitrate, in the treatment of chronic anal fissure [50]. In 1994, **Peter Loder** et al. first proposed the “reversible chemical sphincterotomy” through the application of glyceryl trinitrate. They demonstrated, through manometric studies, the effect of reducing the tone of the anal sphincter at the same level of a lateral sphincterotomy and noticed an increase in perfusion through vascular dilatation [51]. The side effects of the treatment were limited to headache; however, a high rate of recurrence was observed [15,32,52].

An alternative was identified in the local application of calcium channel blockers as they presented fewer side effects [32,53]. In 1999, **Carmine Antropoli** published the first experience with the application of nifedipine [53], and **Emin Carapeti** with diltiazem in 2000 [54]. These drugs rapidly became popular, and diltiazem is currently recommended as a first-line pharmacological treatment for anal fissure by the American Society of Colon and Rectal Surgeons and the Association of Coloproctology of Great Britain and Ireland. [54,55].

### 3.3. 21st Century

In the twenty-first century, several new techniques were proposed for the treatment of chronic anal fissure, as subsequently described.

In addition to botulinum, the infiltration of a different paralyzing toxin, gonyautoxin, a paralyzing phytotoxin produced by dinoflagellates, was tested for the first time in 2005, reporting a good profile of efficacy and safety according to preliminary results [56,57]. However, it has not found widespread use over time.

In 2008, **Gupata** proposed the “closed anal sphincter manipulation” as an improvement to traditional anal dilation in order to obtain the blunt division of internal sphincter fibers [58].

In the same period, another conservative therapeutic alternative was introduced: the “posterior perineal support”, an external mechanical device which, by exerting pressure on the posterior perineum during defecation, reduces the tension of the mucosa at the 6 o’clock position (typical site of the fissure) [59].

Despite the theoretical advantages of these innovations, their use has not spread into common clinical practice.

In 2005, in order to reduce the risks of incontinence, **Dong-Yoon Cho** proposed a “controlled” sphincterotomy, suggesting that the height of the internal sphincter’s section should be selected based on the level of hypertonicity, which are, in ascending order, the section of the sphincter up to the upper edge of the fissure, the section of the sphincter up to the dentate line, and the bilateral section of the sphincter [32].

In 2018, **Mohammed Alawady** et al. proposed posterolateral sphincterotomy with the aim of reducing healing time and postoperative pain in comparison to the lateral approach. Further studies have shown no significant differences between the two techniques, so lateral internal sphincterotomy continues to be the gold standard for the treatment of chronic anal fissure [60].

Moreover, towards the end of the last century, research had begun to understand more deeply the etiology of the pathology. As early as the 1800s, it was known that anal fissures were linked to a hypertonicity of the anal sphincter, but how these two were connected was not fully clarified. In the late 1900s, it was stated that hypertonicity leads to relative ischemia and, consequently, to chronic anal fissure [15,51,61]. Faced with this new perspective, in recent years, new conservative treatments have gradually been proposed that allow healing without the need for a scalpel.

Since 2011, sacral nerve stimulation and posterior tibial nerve stimulation have been proposed as alternatives. Regarding the former, the therapeutic effects are attributed to an increase in blood flow, which consequently improves wound healing [62]. The stimulation of the posterior tibial nerve has the same effect by stimulating the sacral plexus with retrograde pulses. Data on this practice are promising, but still limited, and lateral internal sphincterotomy currently continues to have better outcomes with a lower recurrence rate [32,63,64].

In recent years, it has also been shown that Autologous Adipose Tissue Transplant can promote the healing of lesions associated with local ischemia. Based on this knowledge, some authors have tried to apply this therapy in the treatment of chronic anal fissure with promising results. However, further studies are needed to verify the effectiveness of this technique and its role in comparison with sphincterotomy [32].

A new minimally invasive sphincterotomy technique was proposed by **Dilip Umakant Pathak**. This is the internal transmucosal sphincterotomy (TMIS), which consists of the isolation of the internal sphincter with retractors and sutures followed by its section through a minimal incision in the mucosa. This technique is technically simple, and in preliminary results, it seems to reduce postoperative discomfort compared to the classic lateral internal sphincterotomy (LIS) with similar therapeutic efficacy [65].

Nowadays, thanks to the effectiveness of medical therapy, the first line of treatment for chronic fissures (in addition to correct dietary habits and lifestyle changes, which are always recommended) consists of the application of topical drugs (calcium channel blockers or glyceryl trinitrate) [49,55]. Botulinum toxin infiltration represents the second line of treatment in case of failure with topical drugs [41,55].

The role of surgery is currently reserved for cases unresponsive to medical therapy. It consists of lateral internal sphincterotomy and its variants (in patients with sphincter hypertonicity) or the anal advancement flap (in patients without hypertonicity) [46,52].

In Table 1, the milestones of anal fissure treatment are summarized.

## Figures and Tables

**Table 1:** Milestones in anal fissure therapy.

Author	Year	Innovation
Celso	35 AD	Warm sitz baths, ointments
Razi, Ibn Sina	900–1020	Laxatives, second line of treatment
Le Monnier	1689	Description of the pathology
Boyer	1826	Open lateral sphincterotomy with involvement of the external sphincter
Recamier	1838	Anal dilatation (manual)
Baladin	1849	Closed lateral subcutaneous sphincterotomy with involvement of the external sphincter
Hirschmann	1914	Anal dilatation (instrumental)
Gabriel	1930	Fissurectomy
Eisenhammer	1951	Lateral open sphincterotomy of the internal sphincter alone
Parks	1967	Lateral internal sphincterotomy through skin incision
Samson and Stewart	1970	Anoplasty
Notaras	1971	Lateral subcutaneous internal sphincterotomy
Jost and Schimrigk	1993	Application of botulin toxin
Loder	1994	Chemical sphincterotomy (glyceryl trinitrate)
Antropoli	1999	Chemical sphincterotomy (nifedipine)
Carapeti	2000	Chemical sphincterotomy (diltiazem)

## Data Availability

The original contributions presented in the study are included in the article, further inquiries can be directed to the corresponding author.
